# Novel genomes and genome constitutions identified by GISH and *5S rDNA* and *knotted1* genomic sequences in the genus *Setaria*

**DOI:** 10.1186/1471-2164-14-244

**Published:** 2013-04-11

**Authors:** Meicheng Zhao, Hui Zhi, Andrew N Doust, Wei Li, Yongfang Wang, Haiquan Li, Guanqing Jia, Yongqiang Wang, Ning Zhang, Xianmin Diao

**Affiliations:** 1 Institute of Crops Sciences, Chinese Academy of Agricultural Sciences, Beijing 100081, China; 2 Institute of Millet Crops, Hebei Academy of Agricultural and Forestry Science, Shijiazhuang 050031, China; 3 College of Life Science, Hebei Normal University, Shijiazhuang 050012, China; 4 Department of Botany, Oklahoma State University, Stillwater, Oklahoma 74078, USA; 5 Institute of Cotton Research, Hebei Academy of Agricultural and Forestry Science, Shijiazhuang 050031, China

**Keywords:** *Setaria*, GISH, Genome constitution, Phylogenetic relationships

## Abstract

**Background:**

The *Setaria* genus is increasingly of interest to researchers, as its two species, *S. viridis* and *S. italica*, are being developed as models for understanding C4 photosynthesis and plant functional genomics. The genome constitution of *Setaria* species has been studied in the diploid species *S. viridis*, *S. adhaerans* and *S. grisebachii,* where three genomes A, B and C were identified respectively. Two allotetraploid species, *S. verticillata* and *S. faberi,* were found to have AABB genomes, and one autotetraploid species, *S. queenslandica,* with an AAAA genome, has also been identified. The genomes and genome constitutions of most other species remain unknown, even though it was thought there are approximately 125 species in the genus distributed world-wide.

**Results:**

GISH was performed to detect the genome constitutions of Eurasia species of *S. glauca*, *S. plicata*, and *S. arenaria*, with the known A, B and C genomes as probes. No or very poor hybridization signal was detected indicating that their genomes are different from those already described. GISH was also performed reciprocally between *S. glauca*, *S. plicata*, and *S. arenaria* genomes, but no hybridization signals between each other were found. The two sets of chromosomes of *S. lachnea* both hybridized strong signals with only the known C genome of *S. grisebachii*. Chromosomes of Qing 9, an accession formerly considered as *S. viridis*, hybridized strong signal only to B genome of *S. adherans*. Phylogenetic trees constructed with *5S rDNA* and *knotted1* markers, clearly classify the samples in this study into six clusters, matching the GISH results, and suggesting that the F genome of *S. arenaria* is basal in the genus.

**Conclusions:**

Three novel genomes in the *Setaria* genus were identified and designated as genome D (*S. glauca*), E (*S. plicata*) and F (S*. arenaria*) respectively. The genome constitution of tetraploid *S. lachnea* is putatively CCC’C’. Qing 9 is a B genome species indigenous to China and is hypothesized to be a newly identified species. The difference in genome constitution and origin of *S. verticillata* and *S. faberi* is also discussed. The new genomes and the genome constitutions of *Setaria* species identified in this report provide useful information for *Setaria* germplasm management, foxtail millet breeding, grass evolution and the development of *S. viridis* and *S. italica as* a new model for functional genomics.

## Background

The grass genus *Setaria* Beauv., a member of the tribe Paniceae, in the subfamily Panicoideae of the Poaceae, has approximately 125 species worldwide in tropical, sub-tropical and temperate regions, including crop and weed species with different life cycles and ploidy levels [1,2]. However, the actual number of species in this genus is confused by the presence of multiple names for some species and multiple species under the same name, as well as overlapping morphological characters both within and between species [3,4]. There are approximately 74 species native to Africa, 25 species in America and the remainder in Eurasia [2]. *Setaria italica* (foxtail millet), a crop that was domesticated more than 10 thousand years ago [5], is still cultivated in China, India, Japan and other countries in more arid and semi-arid regions as a stable food grain, and is used as a forage crop in North America, Africa and Australia [6]. *Setaria glauca* (Weigel) Hubb. (yellow foxtail) is also domesticated and cultivated in India as to complement other food sources [3,4]. Most other species of the genus are problematic weeds for agricultural crop production [2], including *S. verticillata* (L.) Beauv. (bristly foxtail) and *S. faberi* Herrm. (giant foxtail). *S. viridis* (L.) Beauv. (green foxtail) is a wide spread species in Eurasia and is known for its repeated evolution of herbicide resistance in North America farms [7]. In the latest phylogenetic analysis, *Setaria* was found to be polyphyletic, with separate groups correlated by geography rather than the existing sub-generic classification [8].

Evolutionary relationships within *Setaria* remain unclear, even after several molecular phylogenetic studies [8,9]. However, several groups within *Setaria* were shown to be monophyletic, including the close relationship between *S. viridis* and *S. italica.* Numerous studies have shown that the domesticated *S. italica* has been shown to be most likely derived from the wild *S. viridis*, including cytological genetical studies [10], RAPDs [11], RFLPs [12], ISSRs [13] and molecular phylogenetic studies [8,9,14]. Detailed studies of the phylogenetic relationships of *Setaria*, using more than 50 species from all over the world and the *knotted1* and *ndhF* gene markers, found that *Setaria* is polyphyletic, with some species of the New World classified into other genera [8,9].

The basic chromosome number of the genus and its close relatives is x = 9 [15,16], but the genome constitution of the group is so far poorly studied. The diploid genome of *S. italica* (2n = 2x = 18) was designated as genome A by Li et al [10]. Diploid *S. viridis* shares the same A genome as *S. italica,* verified by hybrid fertility and cytogenomic, enzymatic and molecular marker studies [12,14,17-19]. *S. adhaerans* (Forssk.) Link ex Chiov. (2n = 2x = 18) was identified as carrying a distinct genome from genome A (labeled B) by genomic in situ hybridization (GISH) [19]. The genome constitution of the tetraploid *S. faberi* (giant foxtail) and *S. verticillata* (bristle foxtail) was identified as being AABB, with 2n = 4x = 36 [19]. GISH studies also indicated that *S. glauca* bears an unknown genome type that is not related to either the A or B genome [19]. The diploid genome of *S. grisebachii* Fourn. ex Hemsl (2n = 2x = 18) was identified as genome C due to their poor hybridization signals with both A of *S. viridis* and B of *S. adhaerans* by GISH [20]. *S. queenslandica* (Domin) was detected as being the first autotetraploid in the genus, with a genome constitution of AAAA, with 2n = 4x = 36 [20]. The genome constitutions of most other species of the *Setaria* genus remain unknown.

The most recent phylogenetic analysis of the genus using the chloroplast marker *ndhF* shows accessions of *S. faberi* and *S. verticillata* grouping with *S. viridis* and *S. italica*[8]. However, other accessions of *S. verticillata* are placed elsewhere, and the authors suggest that this is caused either by the multiple origins of the polyploid and/or homoplasy in the distinguishing characteristic of the retrorse barbs on the sterile bristles in the inflorescence. An earlier study that used the *knotted1* nuclear marker found both multiple placements of separate accessions of *S. verticillata* as well as multiple placements of copies from single accessions [9]. Benalbdelmouna [19] showed that the genome constitution of both *S. faberi* and *S. verticillata* was AABB, which supports the placement of one gene copy of *S. verticillata* with the A genome species *S. italica* and *S. viridis*, and the other copy elsewhere. In the *ndhF* phylogeny two accessions of *S. verticillata* are placed with *S. adhaerens*, shown by Benalbdelmouna to possess genome B. The relationships of *S. faberi* are less clear, primarily because of insufficient sampling, as the *ndhF* phylogeny only contains a single accession of *S. faberi*, and the *knotted1* phylogeny does not contain that species.

Due to its small genome size, diploid nature and self-fertilization, *S. italica* is becoming a new model for functional and evolutionary studies in the grasses, while *S. viridis* is a model for C4 photosynthesis [21-23]. The release of the genomic sequences of foxtail millet has accelerated the establishment of these model systems [23,24]. Understanding the genetic relationships of *Setaria* genome types will be helpful in managing *Setaria* germplasm, and contribute to our understanding of the evolution history of this group of species. Genomic in situ hybridization (GISH) provides a visual and direct method for investigating genomic composition among species, and is especially useful in elucidating the complex origins of polyploid plants. This technique has been already applied in many groups such as *Triticeae*[25], *Brassica*[26], *Nicotiana*[27], *Andropogon*[28], and *Setaria* species [19,20]. In this report, GISH was applied to chromosome preparations of *Setaria* species of Eurasian origin with unknown genome constitution. The known genome types of A, B and C were used as probes to detect the genome composition of these species and to identify new genomes in the *Setaria* group. To further confirm the results obtained by GISH, *5S rDNA* and *knotted1* gene sequences were analyzed using Bayesian methods to elucidate their phylogenetic relationships. Sequences of *5S rDNA* and *knotted1* genes have already useful in the phylogenetic study of many plant species and of *Setaria* relationship in particular [9,14,27].

## Methods

### Plant material

The three diploid known genomes of *S. viridis* (genome A), *S. adhaerans* (genome B) and *S. grisebachii* (genome C) were used as testers to determine the genome constitution of other diploid and polyloid Eurasian species. The polyploid species examined in this paper include *S. glauca*, *S. parviflora* (Poir.) Kerguelen, *S. palmifolia* (Koen.) stapf, *S. lachnea* (Nees) Kunth, *S. plicata* (L*am*.) T. Cooke and *S. arenaria* (Kitag.). The chromosome number of the accessions studied was identified by squashing root tips, as previously reported [20,29]. The chromosome preparations of at least one sample of each species were hybridized with probes made from the known diploid A, B and C species genomes (Table 1). For phylogenetic analysis with genomic sequences of the *5S rDNA* and *knotted1* genes, more accessions were added (Table 1). One sample of *S. viridis* (Qing 9) appeared morphologically distinct from other accessions and was analyzed along with representative accessions. Plant material used in this study, as well as their polyploid characteristics and geographical origin are listed in Table 1.

**Table 1:** Origin of the samples used, chromosome numbers, genome constitution if known, and the copy numbers of DNA fragments from each accession used for phylogenetic analysis

**Species**	**Origin**	**Accession number**	**Code**	**Ploidy level**	**Genome**	**Copy 5SrDNA**	**Copy knotted1**
S. viridis^*^	China (Hebei)	N033	S. vir-Q24	2n = 2x = 18	AA	1	1
*S. viridis*	Russia	09005	S. vir-W56	2n = 2x = 18	AA	1	1
*S. italica*^*+*^	China (Henan)	00024169	S. ita-Y1	2n = 2x = 18	AA	1	1
*S. italica*	South Africa, (Transvaal)	PI 209909	S. ita-C238	2n = 2x = 18	AA	1	1
Qing 9^*+^ (unidentified species)	China (Hebei)	N011	Qin9	2n = 2x = 18	BB	1	1
S. adhaerans^*+^	Spain	02448	S. adh-W94	2n = 2x = 18	BB	1	1
*S. adhaerans*	Hawaii	25001	S. adh-W41	2n = 2x = 18	BB	1	1
S. grisebachii^*^	Mexico	03001	S. gri-W8	2n = 2x = 18	CC	1	1
S. plicata^*^	China (Kunming)	25001	S. pli-N195	2n = 4x = 36	X(EE)	1	2
S. glauca^*+^	Iowa	04004	S. gla-W12	2n = 4x = 36	X(DD)	1	1
*S. glauca*	Canada	04005	S. gla-W13	2n = 4x = 36	X(DD)	1	1
*S. glauca*	Japan	04002	S. gla-W10	2n = 8x = 72	X(DD)	2	2
*S. glauca*	France	14003	S. gla-W82	2n = 4x = 36	X(DD)	1	1
*S. lachnea*^*+*^	Australia	11001	S. lac-W74	2n = 4x = 36	CCC’C’	1	2
*S. palmifolia*^*+*^	China (Kunming)	26001	S. pal-N193	2n = 6x = 54	X(EE)	3	2
*S. parviflora*^*+*^	Brazil	13002	S. par-W79	2n = 4x = 36	X(DD)	2	2
*S. arenaria*^*+*^	China (Kunming)	27001	S. are-N196	2n = 6x = 54	X(FF)	4	2
*S. verticillata*	France	08006	S. ver-W42	2n = 4x = 36	AABB	2	2
*S. faberi*	Russia	02005	S. fab-W5	2n = 4x = 36	AABB	2	1
*S. faberi*	Japan	02006	S. fab-W7	2n = 4x = 36	AABB	2	2
*S. queenslandica*	Australia	PI 316342	S. que-W89	2n = 4x = 36	AAAA	2	1

**Note:** The materials marked with * was used to be labeled as probe. The materials marked with + was used to make chromosome preparations. All materials were used for phylogenetic analysis. The putative genome constitution X represents an unknown genome constitution and the capital letter in the bracket of the Genome column indicated the novel genomes identified in the paper.

### Chromosome and probe preparation

All seeds were germinated on moistened filter paper at 27°C in Petri dishes until the roots were 2 cm long, and then treated with 2 mM 8-hydroxyquinoline for 2 h at room temperature to accumulate metaphases. After rinsing with distilled water, the whole seedlings were fixed in a mixture of freshly prepared 1:3 glacial acetic and ethanol (100%), and stored at −20°C until use. For DNA probes, plants were grown in autoclaved soil, transferred into an open air field at the seedling stage, and DNA extracted from young leaves for PCR amplification of *5S rDNA* gene and *knotted1* gene fragments and for probe preparation for GISH.

Chromosome preparations were done according to Benabdelmouna et al [30], with some modifications. A single root tip was transferred in a drop of 45% acetic acid onto a clean slide before squashing. After squashing, the slides were then scanned for good chromosome spreads at prophase or metaphase stages with phase contrast microscopy, then slips were removed by immersing slides into liquid nitrogen for 10–15 min, and the air-dried slides were stored at room temperature until use.

### In situ hybridization

For genomic in situ hybridization, total nuclear DNA from *S. viridis* (AA), *S. adherans* (BB), *S. grisebachii* (CC), Qing 9, *S. glauca*-W12, and *S. plicata*, were extracted from young plants and genomic DNA was labeled by the nick translation method with digaoxigenin-11-dUTP, (Roche). The method of GISH followed that of Bisht et al [31] with some modifications. The hybridization mixture (20 μl/per slide) included 100–150 ng DNA probe, 1 ng/μl salmon sperm DNA, 50%(v/v) deionized formamide, 2 × SSC, 0.1% SDS, and 10% dextran sulfate. DNA probes were denatured for 10 min at 95°C, immediately quenched on ice for least 10 min, denatured by immersion in 70% formamide-2 × SSC for 3 min at 80°C, then the chromosome preparations were dehydrated for 3 min each in a graded series of 70%, 90%, and 100% ethanol at −20°C. Probe mix was applied to each air-dried slide and hybridized overnight in a moist chamber at 37°C. After hybridization the slides were washed twice in 2 × SSC at 37°C for 3 min and 4 × SSC at room temperature for 5 min, and then the slides were treated with BSA blocking solution (5% BSA-2 × SSC) for 15 min at 37°C. Immunodetection of digoxigenated probes was carried out with Rhodamine conjugated anti-digoxigenin antibodies (Roche). Slides were then washed two times in 4 × SSC for 5 min at room temperature, chromosomes were counter-stained with DAPI in the antifade buffer (10 mg/ml, blue fluorescence). For visualization, chromosome preparations were analyzed using an Olympus epifluorescence microscope with appropriate filters. GISH hybridization results between Setaria species are list in Table 2.

**Table 2:** **GISH hybridization results between *Setaria *species**

**Probe/Chromosome**	***S.ita-*Y1(c)**	**Qing 9(c)**	***S.adh-*W94(c)**	***S.lac-*W74(c)**	***S.par-*W79(c)**	***S.pal-*N193(c)**	***S.gla-*W12(c)**	***S. are-*N196(c)**	***S. pli-*N195(c)**
S. vir-Q24(p)	***√***	***×***	×^*^	***×***	***×***	***×***	×^*^	***×***	***×***
S. adh-W94(p)	×^*^	***√***		***×***	***×***	***×***	×^*^	***×***	***×***
S. gri-W8(p)	×^*^		×^*^	***√***	***×***	***×***	***×***	***×***	***×***
Qing 9(p)			***√***						
S. gla-W12(p)					***√***	***×***		***×***	***×***
S. pli-N195(p)						***√***		***×***	

**Note**: (1) The p in the brackets following each accession indicates corresponding genomic DNA as probes used in GISH performance.

(2) The c in the brackets following each accession indicates corresponding chromosome preparation used in the GISH experiment.

(3) The √ indicates that positive signal was detected between the two samples in this paper, × indicates that no significant signal was detected between the two samples in this paper. × with superscripts * indicates the result with no significant signal was reported previously [19,20].

### Phylogenetic analysis

Total genomic DNA was extracted from fresh leaves using the CTAB procedure [32]. The *5S rDNA* gene fragments from each sample were amplified using primers designed on conserved regions of the *5S rDNA* sequences from barley and wheat [33] with FP: 5^′^-GGACCTCCTGCGAAGTCCT-3^′^ and RP: 5^′^-CCCATCCGTGTACTACTCTC-3^′^. The PCR conditions were 95°C for 5 min, 32 cycles of 94°C for 55 s, 62°C for 25 s and 72°C for 35 s, followed by a final extension of 72°C for 10 min. The PCR mixture (20 μl) contained 100 ng template DNA, 250 μM of each dNTP (Takara), 0.5 μM of each primer, 2.0 μl of reaction buffer, and 1 unit of Ex Taq DNA polymerase (Takara). The *knotted1* (*kn1*) gene fragments including part of the first intron, the whole sequences of the second intron and the second exon as described by Doust et al [9] were amplified using the primers pair of kn211-402F: 5^′^-TCAGAACTTTTGGCCGTGGGT-3^′^ and kn612-402R: 5^′^-GAGATGGACAGCGAGTTGAGC-3^′^. PCR reactions were denatured for 5 min at 95°C and then followed a step-down procedure where annealing temperature was stepped down from 65°C to 59°C, and then 35 amplification cycles were performed, each cycle including denaturation at 95°C for 30 s, annealing at 57°C for 1 min, primer extension at 72°C for 1 min, and a final extension of 72°C for 10 min. Products were separated by electrophoresis on 1.5% agrose gels, and the major band of each sample was isolated from the gel, cleaned using Qiagen columns, cloned into the PUCm-T or PMD18-T vector system, and then transformed into the DH5a strain of *E. coli.* After identification of recombinant clones, for diploid species, a minimum of two clones were sequenced, for tetraploid species, at least six clones were picked and sequenced. *Kn1* and *5S rDNA* fragments obtained were confirmed by comparison with Genbank. Sequences from the same accession were aligned using DNAMAN, and redundant sequences were deleted. Accession number for each clone has been deposited in Gene Bank and listed in Additional file 1: Table S1. Outgroup sequences [AB023618, DQ351339, JQ947589, X61308] were obtained from Genbank.

Multiple alignments of unique sequences from each sample were carried out using T-Coffee [34-36]. The phylogenetic trees based on *5S rDNA* and *kn1* sequences were analyzed under neighbor joining (Mega version 5.1) [37] and Bayesian approaches (Mr Bayes 3.1.2) [38,39]. Evolutionary models were chosen using jModeltest version 0.1.1 [40,41], and in both cases were a general time reversible model with a 4 category gamma rate model. Neighbor-joining analyses were run under the Maximum-composite likelihood model, with gamma values estimated in jModeltest, and tree support estimated with 1,000 bootstrapped sample sets. Mr Bayes was run using a GTR plus gamma model, with branch length unconstrained, branch length priors set to an exponential distribution with a parameter of 10, and shape parameter priors set to an exponential distribution with a parameter of 10. Bayesian analyses were run for 5,000,000 generations, with 4 chains on each of two nodes. Chains were compared every generation and nodes compared every 1,000 generations. Tracer version 1.5 [42] was used to estimate burnin, and a conservative 1,000,000 generations from the beginning of each run were removed. The remaining trees from each run were combined and a majority rule consensus tree used computed to summarize the data.

## Results

### GISH analysis

#### Genome identification of a novel diploid *Setaria* species

**Figure 1:**
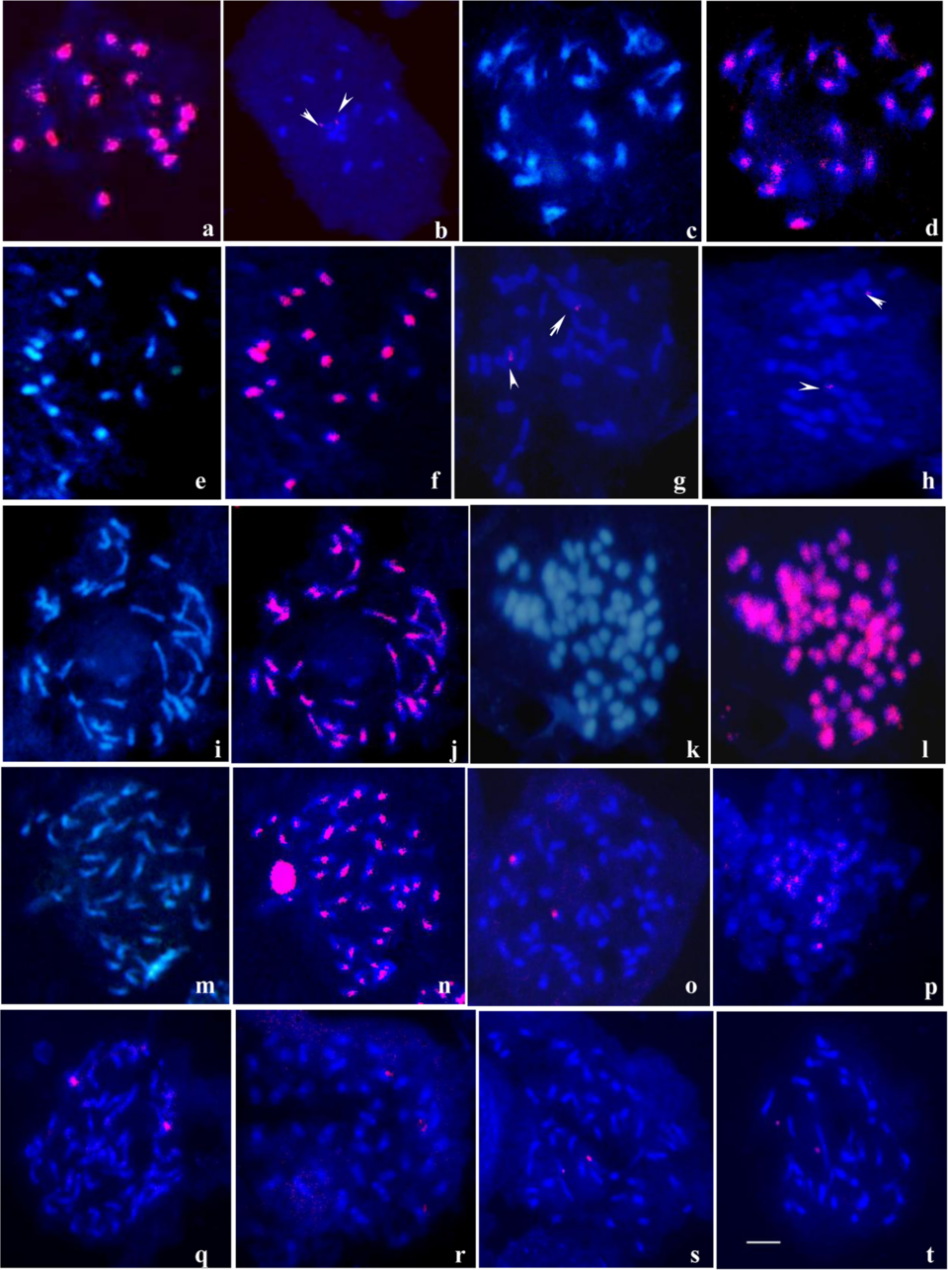
**GISH patterns obtained on different *Setaria *species. **(**a**) GISH was carried out using *S. viridis*-Q24 genomic DNA as probe hybridizing on the chromosome preparation of *S. italica*-Y1. (**b**) Metaphase of Qing 9 probed with *S. viridis*-Q24 total genomic DNA probe, two major spots were detected in the nucleolar organizing regions (arrows). (**c**) DAPI counterstained metaphase plate from *S. adhaerans*-W94. (**d**) The same metaphase plate was hybridized with Qing 9 genomic DNA (red). (**e**) The metaphase of Qing 9 was counterstained with DAPI. (**f**) The same metaphase hybridized with genomic DNA of *S. adhaerans*-W94 (red). (**g**-**h**) Genomic DNA of *S. viridis*-Q24 (**g**) and *S. adhaerans*-W94 (**h**) was applied to *S. lachnea* chromosomes respectively. (**i**) DAPI counterstained metaphase plate from *S. lachnea*. (**j**) The same metaphase hybridized with the total genomic DNA of *S. grisebachii*. (**k**) The metaphase of *S. palmifolia* was counterstained with DAPI. (**l**) The same metaphase plate was hybridized with the genomic DNA of *S. plicata* (red). (**m**) DAPI counterstained metaphase plate from *S. parviflora*-W79. (**n**) The same metaphase hybridized with *S. glauca*-W12 genomic DNA. (**o**-**r**) The metaphases of *S. arenaria* respectively hybridized with the genomic DNA of *S. viridis*-Q24 (**o**), *S. adhaerans*-W94 (**p**), *S. grisebachii* (**q**), and *S. glauca*-W12 (**r**). (**s**) Metaphase of *S. palmifolia* probed with *S. viridis*-Q24 total genomic DNA probe. (**t**) Metaphase plate from *S. glauca*-W12 hybridized with probe from *S. grisebachii*. Bar = 5 μm.

GISH hybridization patterns were investigated using genomic DNA probes to mitotic chromosomes. When the probe prepared from total genomic DNA of *S. virdis*-Q24 was hybridized on the chromosome of *S. italica*, a strong and total painting on all the chromosomes of *S. italica* was observed (Figure 1a). However, when the *S. viridis*-Q24 genomic DNA was used as a probe on chromosome preparations of Qing 9, little hybridization was observed except for two major signal points in the nucleolar organizer region (Figure 1b). When total DNA probe from Qing 9 was applied to chromosome preparations of *S. adhaerans*-W94, all 18 chromosomes strongly hybridized (Figure 1c-d). The same hybridization pattern was obtained in the reciprocal experiment when genomic DNA from *S. adhaerans*-W94 was hybridized to chromosome preparations of Qing 9 (Figure 1e-f).

#### Genome constitution of *S. lachnea*

We performed separate GISH experiments hybridizing chromosome preparations of *S. lachnea* with the three diploid species genomic DNA as probes. When total genomic DNA from *S. viridis*-Q24 (A genome) was used as a probe, no hybridization signals could be detected except for two major signals clustered at pericentromeric regions (Figure 1g), these being regions that are rich in repeat sequences to accumulate hybridization signals. The same patterns were obtained when using B genome of *S. adhaerans*-W94 genomic DNA as probe (Figure 1h). However, a completely different hybridization pattern was obtained when GISH was carried out using genomic DNA of *S. grisebachii* as probe, as all the 36 chromosomes were strongly hybridized with signal painting over most of the length of each chromosome (Figure 1i-j). Although multiple independent hybridizations with different washing protocol with higher stringency were carried out, the same result was obtained and no difference between the two sets of chromosomes was detected (Additional file 2: Figure S1).

#### Genome constitution of *S. glauca* and *S. parviflora*

The genome constitution of *S. glauca*, which is a tetraploid with 2n = 4x = 36 and which was previously identified as being neither A nor B genome by GISH [19], was tested by hybridizing its chromosomes with probes from the genomic DNA of *S. viridis*-Q24, *S. adhaerans*-W94 and *S. grisebachii* (Figure 1t) respectively. All those hybridizations were found to give little or no hybridization signal, except in the pericentromeric regions, which confirms the result obtained by Benabdelmouna et al [19], implying that the two sets of genomes of *S. glauca* are all different from the known genomes of A, B and C.

Chromosome preparations of *S. parviflora*, which is also a tetraploid, were hybridized with probes from the genomic DNA of *S. viridis*-Q24, *S. adhaerans*-W94 and *S. grisebachii* respectively, with the same results obtained as for *S. glauca*. Moreover, the chromosomes of *S. parviflora*-W79 were strongly hybridized with probes from genomic DNA of *S. glauca-*W12 (Figure 1m-n), implying that these two species have a close genetic relationship and they share very similar or the same genome.

#### Genome constitution of *S. plicata* and *S. palmifolia*

Chromosome preparations of the tetraploid species *S. plicata* were hybridized with probes made from the genomic DNA of *S. viridis*-Q24, *S. adhaerans*, *S. grisebachii* and *S. glauca* respectively. All the hybridization gave little or no signals, indicating that the two sets of genomes in *S. plicata* are different from the genome A, B, C and the genome of *S. glauca*. Chromosome preparations of hexaploid species with 2n = 6x = 54 of *S. palmifolia* were also hybridized with genomic DNA probes from *S. viridis*-Q24 (Figure 1s), *S. adhaerans*-W94, *S. grisebachii* and *S. glauca*-W12 respectively and no hybridization signal results were obtained, indicating that the 3 sets of genomes in *S. palmifolia* were quite different from the genomes of A, B, C and that of *S. glauca*. However, when total DNA probes from *S. plicata* were hybridized to chromosomes of *S. palmifolia*, a very strong hybridization pattern distributing all over the chromosomes were obtained (Figure 1k-l), indicating that these two species share the same or similar genome.

#### Genomic constitution of *S. arenaria*

Probes from total genomic DNA of *S. viridis*-Q24 (Figure 1o), *S. adhaerans*-W94 (Figure 1p), *S. grisebachii* (Figure 1q), tetroploid *S. glauca*-W12 (Figure 1r) and *S. plicata* were all hybridized with chromosome preparations of hexaploid species of *S. arenaria (*2n = 6x = 54), but little or no hybridization signal was obtained on the chromosomes. Those GISH hybridizations indicated that the 3 sets of genomes in *S. arenaria* are neither related with the known genomes of A, B and C, nor related with the unknown genomes in species of tetroploid *S. glauca* and *S. plicata*.

### Phylogenetic analysis

#### Phylogeny based on *5SrDNA* sequences

All PCR amplifications for *5S rDNA* sequences from primers designed by D’Hont et al [33] were successful using our *Setaria* genomic DNA as template, and target bands were isolated and subcloned for sequencing. For diploid species, such as *S. viridis*, *S. italica*, *S. adhaerans* and *S. grisebachii*, single sequences were obtained from a sample accession, but for polyploid species, such as *S. verticillata*, *S. faberi*, *S. glauca*, *S. parviflora*, *S. palmifolia*, *S. plicata* and *S. arenaria*, multiple isolates were sequenced and sequences obtained were first aligned to delete redundant sequences, before the unique ones were used for phylogenetic tree construction. The number of unique sequences obtained varied between the different polyploid species (Table 1).

**Figure 2:**
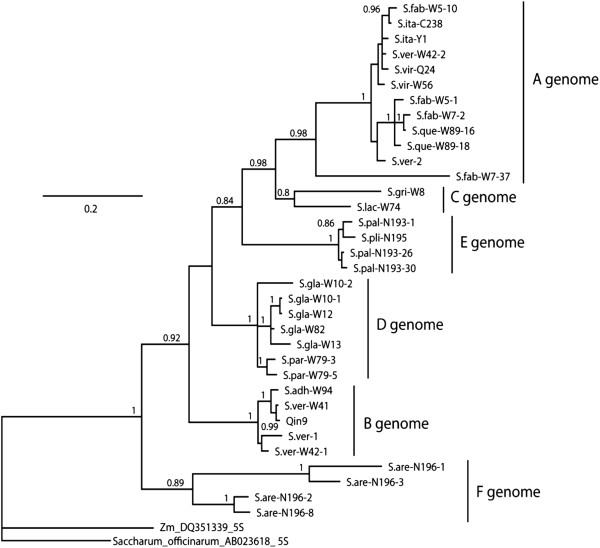
**Bayesian analyses for 5S rDNA sequences amplified in *Setaria*.** Support value are Bayesian posterior probability. Two sequences deposited in Genbank [GenBank: AB023618, DQ351339] were used as outgroups. In some polyploid accessions, more than one copy that different in sequence were obtained, and they are distinguished in the tree by corresponding clone number following each sequence code.

A consensus tree generated from the Bayesian analysis of the *5S rDNA* sequences is displayed in Figure 2, and six groups can be clearly identified (similar results were found for the neighbor joining tree, although with less resolution). The first group, which corresponds with the A genome, is composed of sequences from *S. viridis*, *S. italica, S. verticillata, S. faberi* and *S. queenslandica*. The sequence of *S. faberi*-W7-37 was somewhat divergent from the others of the A group sequences and also carried a large deletion of 41 bp compared to the others in this group. Group B is composed of sequences from *S. adhaerans*, *S. verticillata*, and Qing 9, which corresponds with the B genome. Two sequences of *S. verticillata* deposited in Genbank [AF227011, AF227012] were separately included in A and B group. Group C is composed of two sequences, one from *S. grisebachii* and the other from *S. lachnea*, which corresponds with the known C genome. Although *S. lachnea* is a tetraploid, only one 5S rDNA was amplified. Sequences in group D were all from two tetraploid species of *S. glauca* and *S. parviflora* with high support, indicating a new genome that is different from the known A, B and C genomes. High support values were also seen in Group E, which is composed of sequences from tetraploid *S. plicata* (one sequence) and hexaploid *S. palmifolia* (3 sequences), indicating another novel genome in the *Setaria* species group. Four unique sequences were obtained from hexaploid *S. arenaria* and they were classified into Group F, which implies yet another new genome in the *Setaria* genus.

#### Phylogeny based on *kn1* sequences

**Figure 3:**
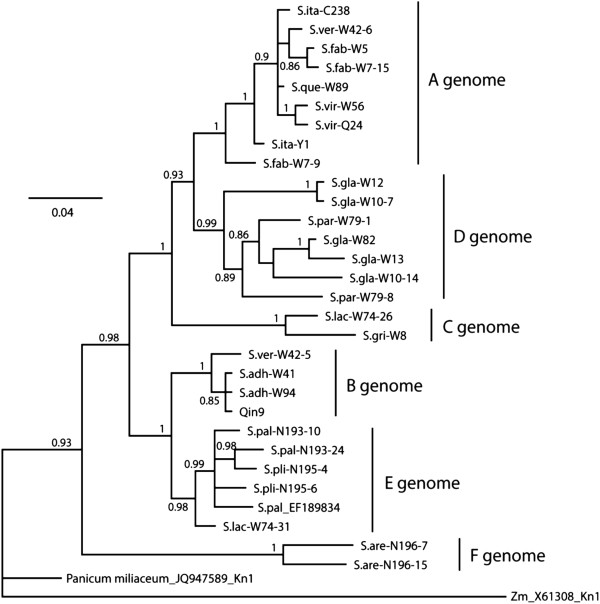
**Bayesian analyses for *kn1 *sequences amplified in *Setaria*.** Support values are Bayesian posterior probability. Two sequences deposited in Genbank [GenBank: JQ947589, X61308] are used as outgroup. In some polyploid accessions, more than one copy that different in sequence were obtained, and they are distinguished in the tree by the corresponding clone number following each sequence code.

Twenty-nine *kn1* unique sequences were included in the phylogenetic analysis, obtained from the samples we used as well as one from *S. palmifolia* in GenBank [EF189834]. The consensus Bayesian tree in Figure 3 was similar in the groups that were delimited in the 5S tree (and similar results were found for the neighbor joining tree, although with less resolution). Group A was composed of the A genome sequences from 5 species, which include *S. viridis*, *S. italica, S. verticillata, S. faberi* and *S. queenslandica*. Group B was made up of 4 sequences corresponding with the known B genome from *S. adhaerans*, *S. verticillata*, and Qing 9, however, we did not detect any sequences from *S. faberi* in this group, albeit its tetraploid AABB genome constitution [19]. For the known C genome only two sequences were amplified from *S. grisebachii* and *S. lachnea* respectively, which form Group C in the tree constructed. However, one sequence from *S. lachnea* (W74-31) was aligned closely to the E group clade with high support. All sequences of *kn1* from *S. glauca* and *S. parviflora* were classified into Group D, which agrees with the GISH result that *S. glauca* and *S. parviflora* share the same or similar genomes. Two sequences of *kn1* from *S. plicata*, two sequences from *S. palmifolia* and the accession of *S. palmifolia* from GenBank [EF189834] were aligned into Group E, which also agrees with the GISH result and *5S rDNA* tree. Only two unique sequences of *kn1* were amplified from the hexaploid *S. arenaria*, which forms Group F in the phylogenetic tree, and their clear differences from other *kn1* sequences is further evidence that the corresponding genomes in *S. arenaria* are distinct from the known A, B, C genomes and from the *S. glauca* and *S. palmifolia* genomes.

Differences in the relationships between the genome types are evident by comparison of the *5S rDNA* and *kn1* trees. In particular, the relationships of genome E (*S. plicata*, *S. palmifolia*) is strongly supported as being with genome B in the *kn1* tree but with genome A and C (albeit with less support) in the *5S rDNA* tree. Likewise, in the *kn1* tree genomes A and D are strongly supported as a phylogenetic group whereas in the *5S rDNA* tree genome D is sister to a clade of A, C, and E genome species. In both trees, genome F (*S. arenaria*) is basal.

## Discussion

### Novel genomes identified by GISH and phylogenetic trees in the genus *Setaria*

There are over 125 species in the genus of *Setaria*[2], yet differences in genome constitution have been poorly studied. The genome of diploid species of green foxtail and foxtail millet was first designated as A genome by Li et al [10], and the genomes of diploid *S. adhaerans* and *S. grisebachii* were designated as B and C genomes respectively [19,20]. The genome constitutions of two tetraploid species, *S. verticillata* and *S. faberi*, were identified being AABB by GISH [19], and that of *S. queenslandica* as AAAA using GISH in our lab [20]; *S. queenslandica* being the only autotetraploid found in the *Setaria* genus. So far no other reports on genomes or genome constitution of *Setaria* species have been published.

Previous reports by GISH identified that the genomes in *S. glauca* were neither A nor B [19], and our result in this report from both GISH and phylogenetic trees indicates that genomes in *S. glauca* were also quite distinct from the newly identified C genome, suggesting a new genome in *S. glauca* which we designated as genome D. A close relationship between *S. glauca* and *S. parviflora* was identified by GISH and phylogenetic trees, imply that they share the D genome. Little or no signals were detected when chromosomes of *S. plicata* and *S. palmifolia* were hybridized with probes from genomic DNA of genomes A, B, C and that of *S. glauca*, and this is supported by the phylogenetic analyses that also show that the sequences from *S. plicata* and *S. palmifolia* are clearly distinguished from those of the known genomes and that of *S. glauca* and *S. parviflora*. This indicates another new genome in *Setaria*, designated as genome E, shared by *S. plicata* and *S. palmifolia*. The GISH pattern and phylogenetic result also indicate that the three sets of genomes in *S. arenaria* are distantly related with the known genome of A, B, C and that of *S. glauca* and *S. plicata*, suggesting another new genome in *S. arenaria,* designated genome F. Thus three new genomes are suggested by combined data from GISH and phylogenetic analyses, although the corresponding species of these new genomes are all polyploid. So far six distinct genome-types coexist in the genus of *Setaria*, reflecting the diversified and diverged genetic composition of this group of grasses, and in good concordance with ISSR result [13] and phylogenetic analyses [8,9]. *Setaria* is suspected to be polyphyletic, based on phylogenetic analysis using both chloroplast and nuclear markers [8,9], so it will be interesting to know whether related taxa outside the various groups of *Setaria* studied here share these genome types.

### The B genome in *S. faberi* is distinct from the B genome in *S. verticillata*

*S. verticillata* and *S. faberi* were all identified as being allotetraploids with a genome constitution of AABB [19]. However, the two primer pairs amplifying the *5S rDNA* and *kn1* gene fragments only amplified from both the B genome diploid of *S. adhaerans* and the B genome of allotetraploid of *S. verticillata*, but gave no amplification from the putative B genome in *S. faberi* (Figures 2 &3), even though multiple PCR amplifications were tried and multiple clones sequenced. The same results were also obtained by Benabdelmouna et al [14] for the 5S rDNA marker. This suggests that the B genome in *S. faberi* has either diverged from the B genome of *S. adhaerans* and *S. verticillata*, or further recombination or backcrossing has occurred that led to the loss of those gene copies*.* Analysis of more molecular markers and a greater number of accessions will be necessary to distinguish between these possibilities. *S. adhaerans* and *S. verticillata* are both characterized by bristles with retrorse barbs, but the bristles of *S. faberi* are the common straight ones. *S. verticillata* appears to have originated from genome duplication of a hybrid between an A genome diploid and a B genome diploid, and the B genome diploid is genetically closely related with the current *S. adhaerans*. The origin of *S. faberi* was from genome duplication of a hybrid between an A genome diploid and an unknown diploid that are relatively distantly related to *S. adhaerans* B genome. Previous hypotheses have suggested that *S. faberi* originated from *S. italica* x *S. adhaerens*, based on the presence of large seeds [43]. Our results do not specifically address this hypothesis, but we suspect that large seeds in *S. italica* are the result of human selection during the last 10,000 years, and that *S. faberi* is of older origin than this. However, genetic diversity analysis of *S. faberi* accessions show little genetic variation [44], indicating that this taxon requires more study.

### Genome constitution of *S. lachnea* was probably CCC’C’

Our GISH experiments clearly show that the two sets of chromosomes of *S. lachnea* had strong hybridization signals covering the entire chromosomes with probe from genomic DNA of diploid *S. grisebachii*, suggesting that *S. lachnea* is probably an autotetroploid species with genome constitution of CCCC (Figure 1i-j). To confirm this conjecture, multiple independent hybridizations with different washing protocol with higher stringency were carried out, giving the same results, and no differences between the two sets of chromosomes were detected (Additional file 2: Figure S1). However, only one of the two unique sequences of *kn1* from *S. lachnea* was closely related to *S. grisebachii* representing the C genome, while the other (W74-31) was more closely related to the E genome sequences (Figure 3). We did not test hybridization of E genome probes to *S. lachnea*, so it remains a possibility that the second genome is related to the E genome. The evidence suggests that one genome set of *S. lachnea* is C, and the other set is a genome closely related with C with some sequence divergence, thus we describe the genome constitution of *S. lachnea* as CCC’C’.

### Qing 9 is probably a newly identified species in B genome of *Setaria*

Qing 9 is a *Setaria* accession collected from Qiema of Luancheng, Shijiazhuang, Hebei province in China in 2003, and treated as a *S. viridis* sample. However, its morphology is clearly distinct from that of *S. viridis,* and ISSR data also indicated that it was distantly related with *S. viridis* samples with A genome, but closely related with B genome species *S. adhaerans*[13]. Our GISH results show that the chromosomes of Qing 9 hybridized well with probe from the B genome *S. adhaerans*, whereas no signal was detected when using the A genome *S. viridis* as probe. This clearly indicated that it is a B genome species and that it was misclassified as a *S. viridis* sample. The *5S rDNA* and *kn1* gene phylogenetic trees also clearly show that Qing 9 is closely related with *S. adhaerans* and distantly related with *S. viridis*, which well support our conjecture. So, Qing 9 is clearly a B genome diploid sample.

Morphological observations also clearly distinguishes Qing 9 from *S. adhaerans.* As a typical B genome species, *S. adhaerans* is characterized with bristles of deep retrorse barbs, tall and slim stem, and narrow leaf blade. But Qing 9 is characterized with shorter and sturdy stem, wide leaf blade, and especially its bristles are weakly retrorse hooked, which is not only clearly different from the deep retrorse hooked *S. adhaerans* but also clearly different from the straight ones of *S. viridis.* Qing 9 does not correspond to any of the twenty three indigenous species described in the Flora of China [45], suggesting that it is a newly identified species. Combine all those data, we make the hypothesis that Qing 9 is probably a not yet detected resident species in China. *S. adhaerans* is a sub-tropical B genome diploid species found around the Mediterranean area (including South Europe and North Africa), thus the identification of novel B genome diploid species indigenous to China will be important for the evolutionary study of the *Setaria* group and the grass family. Detailed studies of this taxon are currently under way.

## Conclusions

The combined data provided by the GISH and phylogenetic analysis indicates that the diploid genome constitutions containing in the polyploid species *S. glauca*, *S. plicata*, and *S. arenaria* are clearly different from the known genomes of *S. viridis* (A), *S. adherans* (B) and *S. grisebachii* (C). These new genomes were designated as genome D, E and F respectively. The genome constitution of *S. lachnea* is probably CCC’C’, and Qing 9 is a B genome species indigenous to China. The results obtained provide useful information for *Setaria* germplasm management, foxtail millet breeding, grass evolution and the development of *S. viridis* and *S. italica as* a new model for functional genomics.

## Competing interests

The authors declare that they have no competing interests.

## Authors’ contributions

XD designed the study and supervised the experiment. HZ, WL, YW and HL participated in the growing of all the materials used. MZ, YW and NZ performed the GISH and sequence cloning. MZ, AND, JG and XD analyzed the Data and drafted the manuscript. All authors discussed the results and conclusion and read and approved the final manuscript.

## Supplementary Material

Additional file 1: Table S1 Each clone was added to accession number deposited in Gene Bank. Click here for file

Additional file 2: Figure S1 Hybridization between *S. grisebachii* and *S. lachnea* with high stringency. The stringency was strengthened by additional wash of 0.1 × SSC at 37°C for 5 min each, 2 × SSC at 37°C for 5 min two times, the genome of S. *grisebachii* hybridized well with the two sets of chromosome of *S. lachnea*. Bar = 5 μm. Click here for file
