# Supplementary material for: Novel genomes and genome constitutions identified by GISH and 5S rDNA and knotted1 genomic sequences in the genus Setaria
Source: BMC Genomics. 2013 Apr 11;14:244. doi: 10.1186/1471-2164-14-244 (PMC3635993; doi:10.1186/1471-2164-14-244)
Supplement: Additional file 1: Table S1 — Each clone was added to accession number deposited in Gene Bank. [file 1471-2164-14-244-S1.doc]

**Additional file 1**

**Table S1. Each clone was added to a**ccession number deposited in Gene Bank

| Gene | Clone Number | Accession Number  in Gene Bank | Gene | Clone Number | Accession Number  in Gene Bank |
| --- | --- | --- | --- | --- | --- |
| Kn1 | S.ita-C238 | KC525370 | 5s rDNA | S.are-N196-2 | KC525401 |
| Kn1 | S.gla-W12 | KC525371 | 5s rDNA | S.are-N196-3 | KC525402 |
| Kn1 | S.ver-W42-5 | KC525372 | 5s rDNA | S.are-N196-8 | KC525403 |
| Kn1 | S.ver-W42-6 | KC525373 | 5s rDNA | S.fab-W5-1 | KC525404 |
| Kn1 | S.que-W89 | KC525374 | 5s rDNA | S.fab-W5-10 | KC525405 |
| Kn1 | S.pal-N193-10 | KC525375 | 5s rDNA | S.fab-W7-2 | KC525406 |
| Kn1 | S.pal-N193-24 | KC525376 | 5s rDNA | S.fab-W7-37 | KC525407 |
| Kn1 | S.pli-N195-4 | KC525377 | 5s rDNA | S.gla-W10-2 | KC525408 |
| Kn1 | S.pli-N195-6 | KC525378 | 5s rDNA | S.gla-W10-1 | KC525409 |
| Kn1 | S.are-N196-7 | KC525379 | 5s rDNA | S.gri-W8 | KC525410 |
| Kn1 | S.are-N196-15 | KC525380 | 5s rDNA | S.ita-C238 | KC525411 |
| Kn1 | S.fab-W5 | KC525381 | 5s rDNA | S.ita-Y1 | KC525412 |
| Kn1 | S.fab-W7-9 | KC525382 | 5s rDNA | S.lac-W74 | KC525413 |
| Kn1 | S.fab-W7-15 | KC525383 | 5s rDNA | S.pal-N193-1 | KC525414 |
| Kn1 | S.gla-W10-7 | KC525384 | 5s rDNA | S.pal-N193-26 | KC525415 |
| Kn1 | S.vir-W56 | KC525385 | 5s rDNA | S.pal-N193-30 | KC525416 |
| Kn1 | S.lac-W74-26 | KC525386 | 5s rDNA | S.par-W79-3 | KC525417 |
| Kn1 | S.lac-W74-31 | KC525387 | 5s rDNA | S.par-W79-5 | KC525418 |
| Kn1 | S.par-W79-1 | KC525388 | 5s rDNA | S.pli-N195 | KC525419 |
| Kn1 | S.par-W79-8 | KC525389 | 5s rDNA | S.gla-W82 | KC525420 |
| Kn1 | S.gla-W82 | KC525390 | 5s rDNA | S.que-W89-16 | KC525421 |
| Kn1 | S.adh-W94 | KC525391 | 5s rDNA | S.que-W89-18 | KC525422 |
| Kn1 | S.ita-Y1 | KC525392 | 5s rDNA | S.ver-W42-1 | KC525423 |
| Kn1 | S.adh-W41 | KC525393 | 5s rDNA | S.ver-W42-2 | KC525424 |
| Kn1 | S.gri-W8 | KC525394 | 5s rDNA | Qin9 | KC525425 |
| Kn1 | Qin9 | KC525395 | 5s rDNA | S.vir-Q24 | KC525426 |
| Kn1 | S.vir-Q24 | KC525396 | 5s rDNA | S.vir-W56 | KC525427 |
| Kn1 | S.gla-W10-14 | KC525397 | 5s rDNA | S.gla-W13 | KC525428 |
| Kn1 | S.gla-W13 | KC525398 | 5s rDNA | S.gla-W12 | KC525429 |
| 5s rDNA | S.adh-W94 | KC525399 | 5s rDNA | S.adh-W41 | KC525430 |
| 5s rDNA | S.are-N196-1 | KC525400 |  |  |  |
