# Supplementary material for: Novel genomes and genome constitutions identified by GISH and 5S rDNA and knotted1 genomic sequences in the genus Setaria
Source: BMC Genomics. 2013 Apr 11;14:244. doi: 10.1186/1471-2164-14-244 (PMC3635993; doi:10.1186/1471-2164-14-244)
Supplement: Additional file 2: Figure S1 — Hybridization between S. grisebachii and S. lachnea with high stringency. The stringency was strengthened by additional wash of 0.1 × SSC at 37°C for 5 min each, 2 × SSC at 37°C for 5 min two times, the genome of S. grisebachii hybridized well with the two sets of chromosome of S. lachnea. Bar = 5 μm. [file 1471-2164-14-244-S2.doc]

**Additional file 2**

**Figure S1. Hybridization between *S. grisebachii* and *S. lachnea* with high stringency.**

The stringency was strengthened by additional wash of 0.1×SSC at 37°C for 5 min each, 2×SSC at 37°C for 5 min two times, the genome of *S. grisebachii* hybridized well with the two sets of chromosome of *S. lachnea*. Bar=5μm


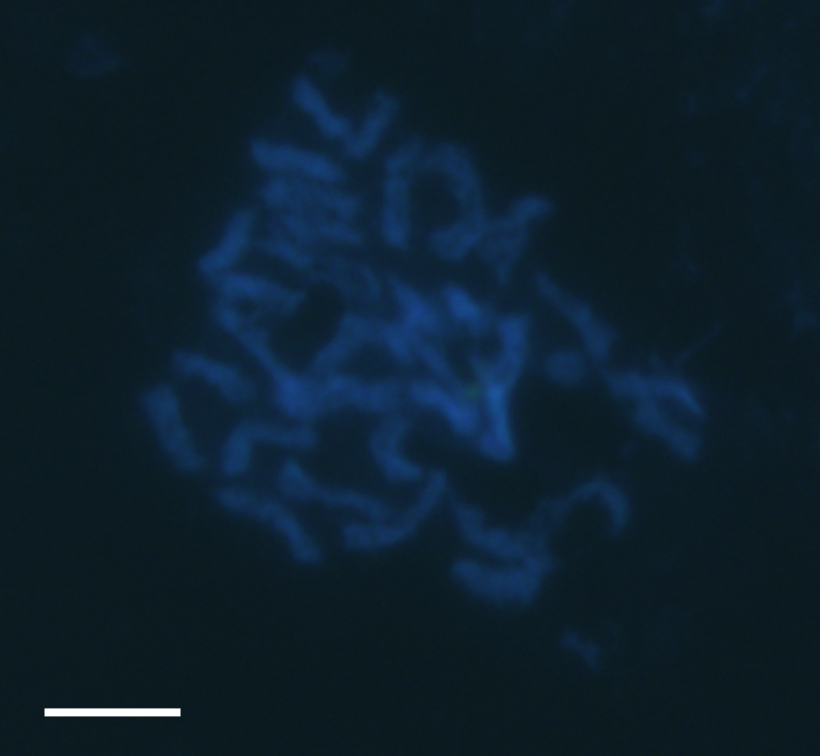


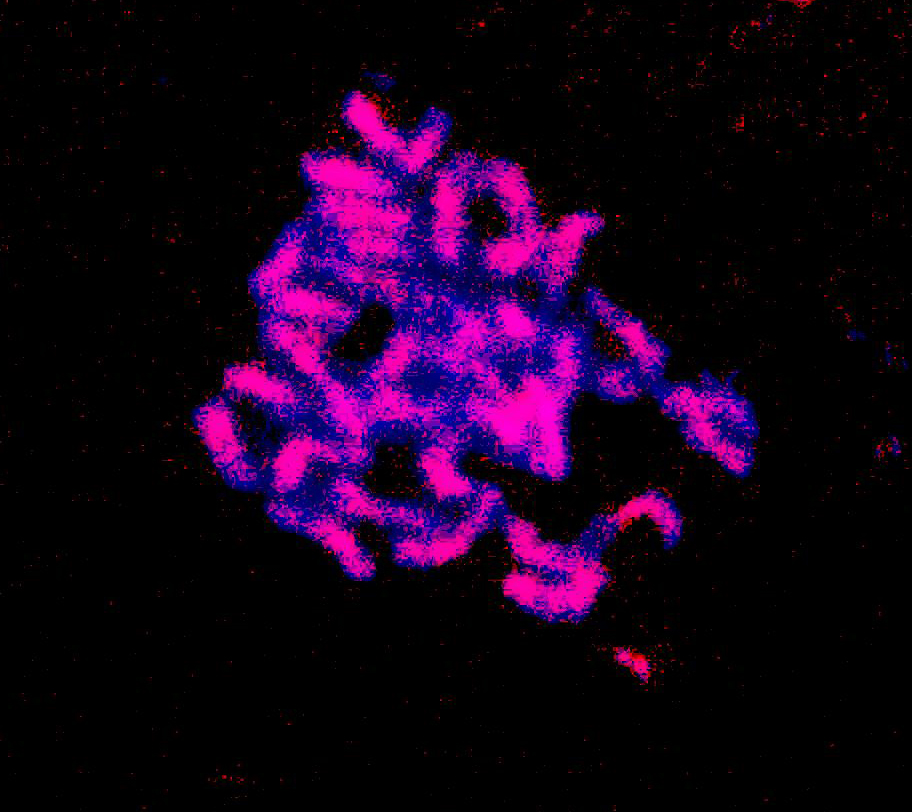


b

a


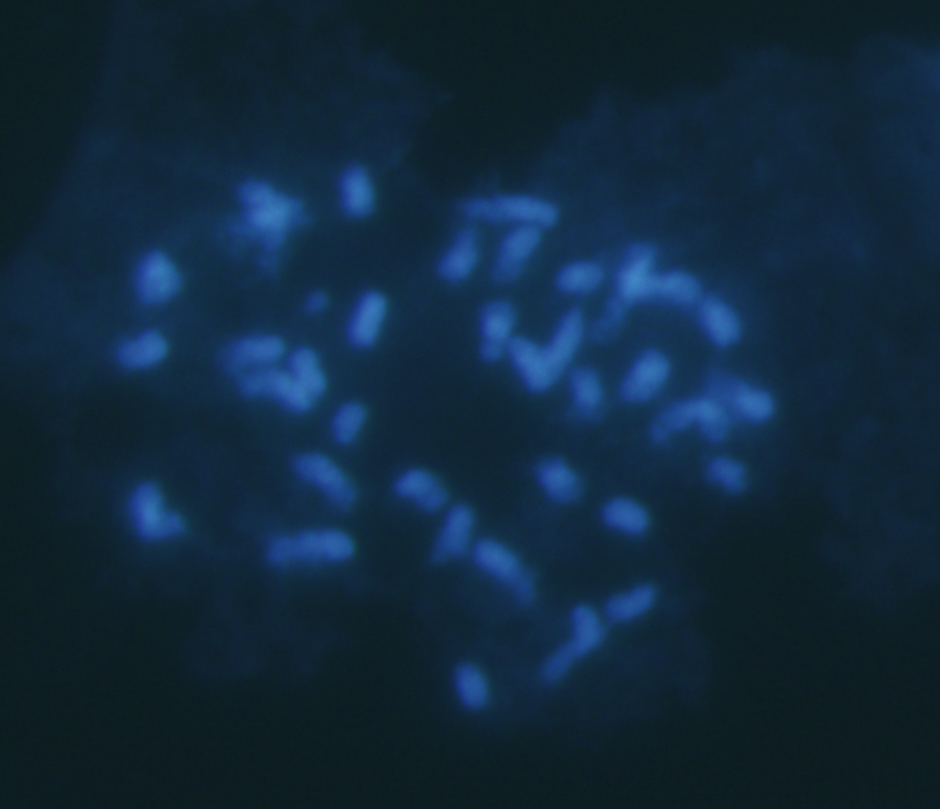


c


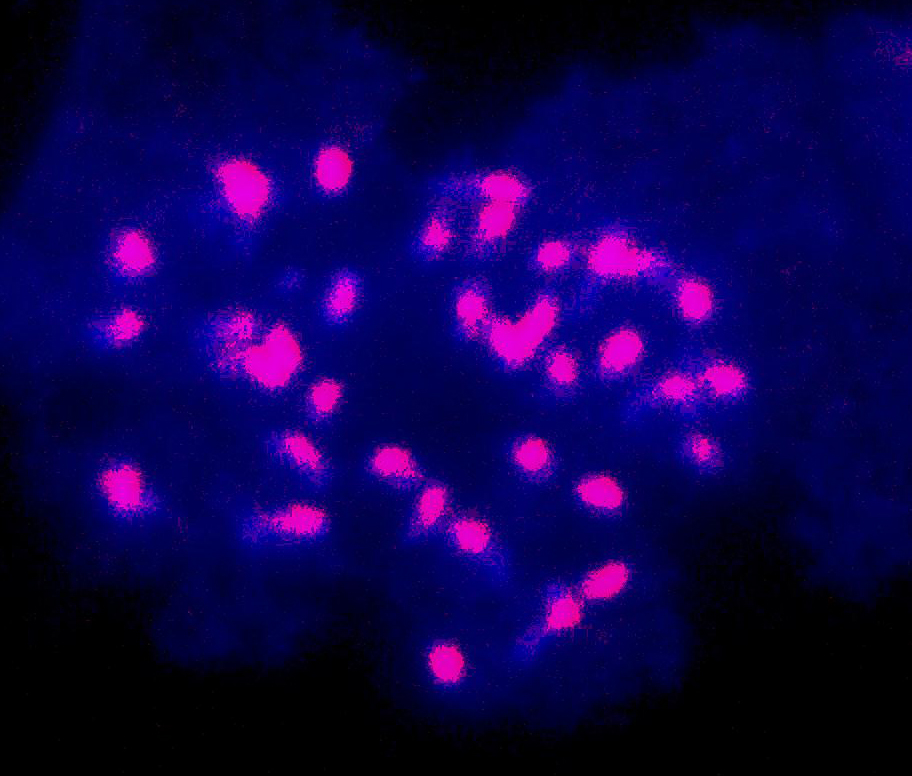


d
